# Outcomes of Transcatheter Edge-to-Edge Repair in Patients With Chronic Kidney Disease: A Retrospective National Inpatient Sample Study

**DOI:** 10.7759/cureus.57420

**Published:** 2024-04-01

**Authors:** Shreyas Singireddy, Stanley E Atencah, Samuel K Dadzie, Kwasi A Opare-Addo, Christopher Chinnatambi

**Affiliations:** 1 Internal Medicine, Piedmont Athens Regional Medical Center, Athens, USA

**Keywords:** hemodialysis, mitral valve, cardiovascular, transcatheter edge-to-edge repair, chronic kidney disease (ckd)

## Abstract

Background

The burden of mitral regurgitation is high, and studies show it is the most common valvular pathology. The management of mitral regurgitation varies and depends on the chronicity, severity, etiology, and comorbidities of the patient. Surgical repair is recommended over replacement. Transcatheter edge-to-edge repair (TEER) has been shown to improve the prognosis of patients with mitral regurgitation and appears relatively safer than surgical repair in patients with high surgical risks. In this study, we examined the outcomes of TEER in patients with chronic kidney disease (CKD) by retrospectively evaluating data from the 2010 to 2016 Nationwide Inpatient Sample (NIS).

Methodology

We retrospectively evaluated data from the 2010 to 2016 NIS. TEER was identified using International Classification of Diseases, Ninth Revision, Clinical Modification (ICD-9-CM) and International Classification of Diseases, Tenth Revision, Clinical Modification (ICD-10- CM) codes of 35.97 and 02UG3JZ, respectively, from our dataset. The study sample was stratified based on renal function into two groups (CKD and no CKD). Patients with CKD were identified using ICD-9-CM codes 585.3, 585.4, 585.5, and 585.6 and ICD-10-CM codes N18.3, N18.4, N18.5, and N18.6.

Results

There was no significant difference in major complications and overall complications between patients with and without CKD. However, heart failure, non-ST-elevation myocardial infarction, cardiac tamponade, and cardiogenic shock were more prevalent in the CKD group. Patients with CKD were also more likely to sustain respiratory failure, post-procedure hemothorax, and require blood product transfusions. For renal complications, analysis showed that superimposed acute kidney injury and the need for hemodialysis were more common in the CKD group. Of note, there was no difference in neurologic complications, gastrointestinal bleeding, and thromboembolic complications between both groups. Patients with CKD who underwent TEER were more likely to have prolonged hospital stays without a significant increase in hospitalization charges. These patients were also more likely to be discharged to rehabilitation facilities.

Conclusions

CKD confers significant morbidity and mortality to patients undergoing TEER. Providers should be aware of these discrepancies in outcomes for patients with CKD in need of TEER to help better optimize their care.

## Introduction

Worldwide, epidemiological studies show that mitral regurgitation is the most common cardiac valvular pathology. It has been estimated to affect close to 2% of the world’s population [1]. The burden of mitral regurgitation is high. Lindmark et al. estimated a 30% five-year mortality risk when compared to patients in the same age-matched control group [2]. Significantly, the five-year mortality risk drops almost to that of the general population when matched according to age if mitral regurgitation is treated successfully [3].

The management of mitral regurgitation varies and depends on the chronicity, severity, etiology, and comorbidities of the patient. Medical and surgical options are the two modalities of treatment. Data have shown no significant evidence of the use of pharmacological agents such as beta-blockers, angiotensin receptor blockers, or angiotensin-converting enzyme inhibitors in the treatment and prevention of the progression of mitral regurgitation [4].

The American College of Cardiology/American Heart Association (ACC/AHA) recommends surgical repair over replacement in most patients when indications are met. In patients who are at high surgical risk, transcatheter edge-to-edge repair (TEER), which is a class 2a recommendation as per ACC/AHA guidelines, has been shown to improve the prognosis of patients with mitral regurgitation [5].

TEER is a percutaneous procedure that utilizes the Mitra-Clip device to approximate and fuse the anterior and posterior leaflets of the mitral valve, subsequently reducing the regurgitant volume. TEER, however, is not without complications [6].

Patients with chronic kidney disease (CKD) remain one of the patient populations with the highest perioperative risks. Among the causes of morbidity and mortality in patients with CKD, cardiovascular causes predominate [7]. TEER appears relatively safer than surgical repair of mitral regurgitation in patients with high surgical risk [5]. Only a handful of studies have reported the outcomes of TEER in patients with CKD. Therefore, our study focuses and explores the outcomes of TEER in patients with CKD.

## Materials and methods

Data sources

We retrospectively evaluated data from the 2010 to 2016 Nationwide Inpatient Sample (NIS), the largest publicly available database of all-payer hospital inpatient stays in the United States [8]. The NIS is a set of longitudinal hospital inpatient databases developed by the Agency for Healthcare Research and Quality for the Healthcare Cost and Utilization Project. The NIS contains data from approximately 8 million hospital stays annually and is designed to approximate a 20% stratified sample of US community hospitals, excluding rehabilitation and long-term acute care hospital units. The NIS allows healthcare researchers to help identify, track, and analyze national trends in healthcare utilization, access, charges, quality, and outcomes with data on all patients, regardless of the payer.

Study population and outcomes

TEER was identified using the International Classification of Diseases, Ninth Revision, Clinical Modification (ICD-9-CM) and International Classification of Diseases, Tenth Revision, Clinical Modification (ICD-10-CM) codes of 35.97 and 02UG3JZ, respectively, from our dataset. Patients younger than 18 years and those with missing demographic data were excluded. The study sample was stratified based on renal function into the following two groups: CKD and no CKD. CKD patients were identified using ICD-9-CM codes 585.3, 585.4, 585.5, and 585.6 and ICD-10-CM codes N18.3, N18.4, N18.5, and N18.6.

Baseline characteristics including CHA₂DS₂-VASc score (ICD-9-CM codes as per Chen et al. and ICD-10-CM codes as per Ueberham et al.), procedural complications (ICD-9/10-CM codes as per Munir et al.), and inpatient outcomes including mortality (reported as a distinct categorical variable in the dataset), length of stay, and hospitalization costs were compared in patients with TEER based on baseline renal function (CKD and no CKD) [9-11]. The prevalence of acute kidney injury (AKI) was also compared between CKD and no CKD TEER groups. We also analyzed the independent association of CKD with outcomes of major complications (defined as the composite of pericardial effusion requiring intervention, cardiac arrest, ischemic stroke/transient ischemic attack, hemorrhagic stroke, systemic embolism, myocardial infarction, and peripheral vascular complications, which included arteriovenous fistula, pseudoaneurysm, access site hematoma, retroperitoneal bleeding, and venous thromboembolism), inpatient mortality, prolonged hospital stay (defined as length of stay >1 day), and increased hospitalization charges (median hospitalization charges >$174,462, inflation-adjusted for 2016 using the Chained Consumer Price Index for all urban consumers and medical care services from the US Bureau of Labor Statistics) [12]. Additionally, factors associated with in-hospital mortality among patients who underwent TEER procedures were analyzed.

Statistical analysis

Descriptive statistics are presented as frequencies with percentages for categorical variables and as median with interquartile range (IQR) for continuous variables. Baseline characteristics were compared using a Pearson chi-squared test for categorical variables and the univariate linear regression test for continuous variables. For crude comparison of procedural complications and in-hospital outcomes among the study groups, the Pearson chi-squared test was used. For assessment of the independent association of CKD with outcomes, including major complications, inpatient mortality, length of stay >1 day, median hospitalization charges >$174,462, and AKI, a single-step survey-based multivariable logistic regression model was used. Age, sex, race/ethnicity, CHA₂DS₂-VASc score, and 29 Elixhauser comorbidities (heart failure, valvular disease, pulmonary circulation disease, peripheral vascular disease, paralysis, neurological disorders, chronic pulmonary disease, diabetes without complications, diabetes with chronic complications, hypothyroidism, hypertension, renal failure, liver disease, peptic ulcer, acquired immunodeficiency syndrome, lymphoma, metastatic cancer, solid tumor without metastasis, collagen vascular disease, coagulopathy, obesity, weight loss, fluid and electrolyte disorders, chronic blood loss anemia, deficiency anemia, alcohol abuse, drug abuse, psychoses, and depression) were used for adjustment. A p-value <0.05 was considered statistically significant. All statistical analyses were performed using the STATA statistical software package (version 16.1; StataCorp LP, College Station, TX, USA). We used the complex survey design of the NIS and applied sample weights, strata, and clusters to generate national estimates.

## Results

A total of 10,415 patients were included in this national inpatient study, of whom 2,618 had CKD, as shown in Table 1. The baseline characteristics of these patients were stratified based on the diagnosis of CKD, as demonstrated in Table 2. Patients undergoing TEER were more likely to be male, 75 years and older, and Caucasian in the CKD versus no CKD groups. Stratification was also done based on underlying comorbid medical conditions and lifestyle, as shown in Table 2. The primary expected payer for patients undergoing TEER was more likely to have Medicare and less likely to have Medicaid or private insurance. In terms of comorbidities, patients with CKD were in the greater proportion likely to have heart failure, valvular disease, peripheral vascular disease, hypertension, diabetes with complications, and renal failure.

**Table 1:** Weighted counts representing national estimates. CKD = chronic kidney disease; TEER = transcatheter edge-to-edge repair

	2010–2016	2010	2011	2012	2013	2014	2015	2016
Number of patients who underwent TEER	10,460	97	343	475	520	1,610	3,070	4,345
Number of patients who underwent TEER after exclusion (age <18 and missing gender information)	10,415	97	343	475	510	1,595	3,055	4,340
No CKD	7,797	87	315	400	425	1,145	2,310	3,115
CKD	2,618	10	28	75	85	450	745	1,225

**Table 2:** Baseline characteristics of the study population of patients undergoing TEER stratified based on CKD. CKD = chronic kidney disease; TEER = transcatheter edge-to-edge repair; IQR = interquartile range; AIDS = acquired immunodeficiency syndrome

Variables	No CKD (n = 7,797)	CKD (n = 2,618)	P-value
Age, median (IQR)	79 (69–85)	79 (72–85)	0.122
Age category (years)			
<65	1,337 (17.5)	325 (12.7)	0.0362
65–74	1,304 (17.1)	470 (18.4)	
≥75	4,979 (65.3)	1,758 (68.9)	
Female (%)	3,801 (48.7)	1,049 (40.1)	<0.001
Race (%)			
White	5,838 (80.5)	1,714 (73.8)	<0.001
Black	403 (5.6)	290 (12.5)	
Hispanic	525 (7.2)	145 (6.2)	
Asian or Pacific Islander	185 (2.6)	90 (3.9)	
Other	299 (4.1)	85 (3.7)	
CHA₂DS₂-VASc score (%)			
0	5 (0.1)	0 (0.0)	<0.001
1	229 (2.9)	10 (0.4)	
2	774 (9.9)	120 (4.6)	
3	1,630 (20.9)	330 (12.6)	
4	2,422 (31.1)	775 (29.6)	
5	1,868 (24.0)	930 (35.5)	
≥6	869 (11.1)	454 (17.3)	
Comorbidities (%)			
Congestive heart failure	2,919 (37.4)	1,325 (50.6)	<0.001
Valvular disease	3,749 (48.1)	1,495 (57.1)	<0.001
Pulmonary circulation disorders	1,160 (14.9)	575 (22.0)	<0.001
Peripheral vascular disorders	934 (12.0)	489 (18.7)	<0.001
Paralysis	99 (1.3)	55 (2.1)	0.1721
Neurological disorders	363 (4.7)	140 (5.3)	0.5164
Chronic pulmonary disease	2,075 (26.6)	739 (28.2)	0.4858
Diabetes without complications	1,374 (17.6)	579 (22.1)	0.0185
Diabetes with complications	259 (3.3)	434 (16.6)	<0.001
Hypothyroidism	1,425 (18.3)	476 (18.2)	0.9555
Hypertension (uncomplicated and complicated)	5,577 (71.5)	2,229 (85.1)	<0.001
Renal failure	1,192 (15.3)	2,618 (100.0)	<0.001
Liver disease	204 (2.6)	125 (4.8)	0.0138
Peptic ulcer disease excluding bleeding	25 (0.3)	25 (1.0)	0.0703
AIDS	5 (0.1)	5 (0.2)	0.418
Lymphoma	90 (1.2)	35 (1.3)	0.7003
Metastatic cancer	45 (0.6)	0 (0.0)	0.0828
Solid tumor without metastasis	125 (1.6)	65 (2.5)	0.1935
Rheumatoid arthritis/Collagen vascular disease	368 (4.7)	145 (5.5)	0.4166
Coagulopathy	899 (11.5)	471 (18.0)	<0.001
Obesity	659 (8.5)	315 (12.0)	0.0159
Weight loss	338 (4.3)	194 (7.4)	0.0053
Fluid and electrolyte disorders	797 (19.5)	394 (34.5)	<0.001
Blood loss anemia	84 (1.1)	30 (1.1)	0.9036
Deficiency anemia	1,431 (18.4)	994 (38.0)	<0.001
Alcohol abuse	95 (1.2)	15 (0.6)	0.2149
Drug abuse	64 (0.8)	15 (0.6)	0.5741
Psychoses	71 (0.9)	14 (0.5)	0.4315
Depression	594 (7.6)	175 (6.7)	0.4666
Payer (%)			
Medicare	6,254 (80.4)	2,323 (88.7)	<0.001
Medicaid	274 (3.5)	60 (2.3)	
Private	1085 (13.9)	175 (6.7)	
Self-pay	60 (0.8)	5 (0.2)	
Other	109 (1.4)	55 (2.1)	
Bed size of the hospital (%)			
Small	395 (5.1)	185 (7.1)	0.349
Medium	1,298 (16.6)	390 (14.9)	
Large	6,104 (78.3)	2,043 (78.0)	
Hospital location (%)			
Rural	25 (0.3)	5 (0.2)	0.6027
Urban non-teaching	729 (9.4)	285 (10.9)	
Urban teaching	7,042 (90.3)	2,328 (88.9)	
Median income (quartile, %)			
0–25th percentile	1,708 (22.4)	640 (24.8)	0.5568
26–50th percentile	1,809 (23.7)	574 (22.2)	
51–75th percentile	2,061 (27.0)	729 (28.2)	
76–100th percentile	2,053 (26.9)	640 (24.8)	

As shown in Table 3, there was no significant difference in major complications between both groups. However, heart failure, non-ST-elevation myocardial infarction, cardiac tamponade, and cardiogenic shock were more prevalent in the CKD group. Patients with CKD were also more likely to sustain respiratory failure, hemothorax post-procedure, and require blood product transfusions. For renal complications, our analyses particularly showed that superimposed AKI and the need for hemodialysis were more common in the CKD group. Of note, there was no difference in neurological complications, gastrointestinal bleeding, and thromboembolic complications between both groups.

**Table 3:** Complications in patients undergoing TEER stratified based on CKD. CKD = chronic kidney disease; TEER = transcatheter edge-to-edge repair; CPR = cardiopulmonary resuscitation; STEMI = ST-elevation myocardial infarction; NSTEMI = non-ST-elevation myocardial infarction; PCI = percutaneous coronary intervention; AV = arteriovenous; TIA = transient ischemic attack; GI = gastrointestinal; AKI = acute kidney injury

Variables	No CKD (n = 7,797)	CKD (n = 2,618)	P-value
Overall complications	7,457 (95.7)	2,523 (96.4)	0.4746
Major complications	1,036 (13.3)	365 (13.9)	0.7269
Any cardiovascular complication			
Cardiac arrest/CPR	413 (5.3)	120 (4.6)	0.5714
STEMI	30 (0.4)	0 (0.0)	0.1558
NSTEMI	94 (1.2)	65 (2.5)	0.0343
Heart failure	2,155 (27.6)	1,134 (43.3)	<0.001
Complete heart block	148 (1.9)	49 (1.9)	0.9865
PCI	74 (0.9)	45 (1.7)	0.1523
Mitral or aortic valve disorder	6,684 (85.7)	2,194 (83.8)	0.2768
Pericardial effusion/Hemopericardium	10 (0.1)	5 (0.2)	0.7488
Cardiac tamponade	20 (0.3)	25 (1.0)	0.0342
Pericarditis	15 (0.2)	0 (0.0)	0.3141
Pericardiocentesis	50 (0.6)	15 (0.6)	0.8632
Cardiogenic shock	289 (3.7)	169 (6.5)	0.0064
Left heart catheterization	733 (9.4)	274 (10.5)	0.4657
Any systemic complication			
Anaphylaxis	5 (0.1)	5 (0.2)	0.4164
Arterial thrombosis	31 (0.4)	5 (0.2)	0.4823
Deep venous thrombosis	115 (1.5)	50 (1.9)	0.4936
Septic shock	65 (0.8)	35 (1.3)	0.3068
Any peripheral vascular complication			
AV fistula	10 (0.1)	0 (0.0)	0.4105
Pseudoaneurysm	40 (0.5)	15 (0.6)	0.8705
Local site hematoma	183 (2.3)	105 (4.0)	0.0635
Local site bleeding	125 (1.6)	50 (1.9)	0.6478
Any neurological complication			
Hemorrhagic stroke	30 (0.4)	0 (0.0)	0.1517
Ischemic stroke	155 (2.0)	45 (1.7)	0.6802
TIA	24 (0.3)	5 (0.2)	0.6505
Any gastrointestinal or hematological			
GI bleeding	120 (1.5)	45 (1.7)	0.7667
Retroperitoneal bleeding	20 (0.3)	5 (0.2)	0.7909
Hemothorax after procedure/Unknown	10 (0.1)	20 (0.8)	0.0189
Blood products transfusion	696 (8.9)	400 (15.3)	<0.001
Any pulmonary complications			
Post-procedure/Iatrogenic pneumothorax/Air leak	49 (0.6)	5 (0.2)	0.2292
Pleural effusion	353 (4.5)	169 (6.5)	0.0827
Pneumonia (bacterial)	184 (2.4)	94 (3.6)	0.0923
Pulmonary embolism	45 (0.6)	0 (0.0)	0.08
Respiratory failure	713 (9.1)	354 (13.5)	0.0052
Renal complications			
Hemodialysis	35 (0.4)	389 (14.9)	<0.001
AKI	902 (11.6)	785 (30.0)	<0.001

Patients with CKD who underwent TEER were more likely to have prolonged hospital stays without a significant increase in hospitalization charges. These patients were also less likely to be discharged home (Table 4).

**Table 4:** Hospital outcomes and resource utilization in patients undergoing TEER stratified based on CKD. $ = adjusted for inflation using the Consumer Price Index (CPI) for medical care services in US city average, all urban consumers, chained, with 2016 as the reference point. CKD = chronic kidney disease; TEER = transcatheter edge-to-edge repair; OR = odds ratio; CI = confidence interval; IQR = interquartile range

Variables	No CKD (n = 7,797)	CKD (n = 2,618)	P-value	OR (95% CI)	Adjusted OR (95% CI)
Died at discharge, n (%)	159 (2.0)	95 (3.6)	0.0463	1.81 (1.00–3.28)	1.46 (0.29–7.35)
Home/routine/self-care, n (%)	5,076 (65.1)	1,469 (56.1)	<0.001	0.69 (0.55–0.85)	0.85 (0.50–1.46)
Non-home discharges, n (%)	2,680 (34.8)	1,144 (43.9)	-	-	-
Acute kidney injury, n (%)	902 (11.6)	785 (30.0)	<0.001	3.27 (2.53–4.23)	1.20 (0.64–2.24)
Resource utilization, median (IQR)	-	-	-	-	-
Length of stay, days	3 (1 - 6)	3 (2 - 9)	-	-	-
Prolonged length of stay, 1 day	5,700 (73.1)	2,223 (84.9)	<0.001	2.07 (1.55–2.76)	2.07 (0.99–4.32)
Total charges of hospitalization, $	173,904 (117,899 - 247,645)	178,499 (125,958–269,951)	-	-	-
Total charges of hospitalization > median $174,462, n (%)	3,825 (49.5)	1,320 (51.4)	0.4997	1.08 (0.87–1.35)	0.74 (0.43–1.28)

## Discussion

We report the following key findings in our large real-world population study of outcomes of TEER in patients with and without CKD. First, there was a higher prevalence of comorbidities among patients undergoing TEER who had CKD compared to those without CKD. Second, the presence of CKD was associated with an independently increased risk of post-procedural major adverse events and, subsequently, prolonged length of stay and higher costs compared to the non-CKD group. Third, in-hospital mortality was significantly higher among patients with CKD undergoing TEER compared to non-CKD patients.

The prevalence of mitral regurgitation is reported to be higher among patients with CKD compared to patients without CKD. In an observational study involving 78,059 patients, patients with CKD had a prevalence of mitral regurgitation almost twice compared to non-CKD (42.9% vs. 23.8%) [13]. Data from the National Cardiovascular Data Registry Transcatheter Valve Therapy Registry suggests that 77% of patients undergoing transcatheter mitral valve repair in the United States had kidney disease [14]. Baseline CKD is associated with poor long-term outcomes, including mortality and prolonged hospitalization in patients undergoing TEER, regardless of the degree of improvement in glomerular filtration rate following TEER, according to multiple single-center studies [15]. It is, therefore, important to evaluate the outcomes in patients with CKD and compare them to patients without CKD to help select appropriate candidates who will benefit most from the procedure.

Several studies have demonstrated that patients with renal disease undergoing TEER have a significantly higher mortality than those without renal disease [11,16]. In one study involving 5,213 patients undergoing TEER at 204 hospitals, pre-procedural renal disease, particularly stage 3 and 4 CKD, was associated with one-year mortality observed in nearly one-third of the population [16]. Indeed, the direct correlation between the severity of CKD and the risk of mortality after TEER was replicated in another study where the mortality rate at one year was 15.0% for the overall CKD cohort. When stratified by stage of CKD, mortality rates were found to be 9.0%, 20.6%, and 26.0% among patients with CKD stages 1 or 2, stage 3, and stages 4 or 5, respectively [17]. These findings are consistent with our study which showed a higher mortality in patients with CKD undergoing TEER.

An observational study of 15,260 patients from the NIS database found that on unadjusted analyses, cardiogenic shock was more prevalent after TEER in patients with end-stage renal disease (ESRD) than in non-ESRD patients (7.7 vs. 4.3%, p < 0.01). On adjustment, however, this difference became insignificant (10.0% vs. 7.7%, p < 0.16). This result may have been due to the exclusion of patients with other stages of CKD in the ESRD arm of the study [14]. Sawalha and colleagues, however, in a study involving 4,645 patients, demonstrated a significantly higher prevalence of cardiogenic shock among patients with CKD after TEER compared to those without (odds ratio (OR) = 1.99; 95% confidence interval (CI) = 1.14-3.46) [18]. This is similar to our result which demonstrated a higher rate of cardiogenic shock among the CKD cohort compared to non-CKD patients (6.5% vs. 3.7%, p<0.007). This significant finding may be explained by the increased risk and prevalence of bleeding observed in renal disease patients.

Shah et al. demonstrated in their study that patients with creatinine clearance ≤30 mL/minute not on dialysis had significant readmission rates because of heart failure at one-year follow-up (OR = 1.73; 95% CI = 1.33-2.25, p < 0.001) [16]. In another study involving analyses of data from a multicenter registry, there was no significant difference between the advanced CKD group, moderate CKD group, and non-CKD groups in terms of readmissions due to heart failure at one month and six months (40.0% vs. 21.0% vs. 18.3%, p = 0.10).

In our study, patients with CKD were more likely to receive blood product transfusions compared to the non-CKD group (15.1% vs. 9.0%, p < 0.001). These results are similar to those found by multiple other studies. In one study, 19.1% of patients without CKD undergoing transcatheter mitral valve replacement experienced bleeding episodes requiring blood transfusions while 25% of patients with CKD required blood transfusions [19]. Shah et al. again demonstrated that patients with ESRD were more than twice as likely to be transfused after a TEER compared to patients without ESRD (14.1% vs. 6.6 %, p < 0.01) [14]. This finding may be related to the increased risk of bleeding in patients with CKD due to platelet dysfunction effect on the coagulation cascade [16].

Concerning respiratory complications in patients with CKD undergoing transcatheter edge-to-edge mitral valve repair, respiratory failure was the most concerning finding in this group. There was, however, no significant difference in the occurrence of post-procedural pneumothorax, pulmonary embolism, bacterial pneumonia, and pleural effusion among patients with CKD and the rest of the population. This could be due to the fewer known pulmonary complications compared to more common cardiovascular complications such as cardiac tamponade and vascular injuries [20]. Patients with CKD were also more likely to develop iatrogenic hemothorax after TEER. Local complications from peripheral venous access have been reported which include hematoma formation, pseudoaneurysm, arteriovenous fistula, and retroperitoneal hemorrhage [21]. None of these were particularly more prevalent in the CKD cohort undergoing TEER, though patients with CKD were more likely to undergo transfusion with blood products. Doulamis et al. established an increased incidence of AKI post-TEER for mitral regurgitation, especially in patients with underlying CKD. They believed that most of the age group undergoing the procedure had some underlying kidney impairment from co-existing chronic diseases like hypertension and diabetes [22]. In fact, approximately 6 out of 10 patients in their study were found to have advanced CKD at baseline, with the greatest predictor of AKI corresponding to CKD stage 4. This likely explains the high rates of AKI in CKD patients post-TEER and their need for hemodialysis compared to those without CKD. AKI directly influenced prolonged hospitalization and mortality, which agreed with Kalbacher et al. [23].

Lastly, there is increased resource utilization and healthcare costs associated with patients with CKD undergoing TEER due to prolonged hospital stay and discharge disposition, compared to those without CKD, but not necessarily an increase in direct hospitalization charges. Patients without CKD were more likely to be discharged home with self-care, whereas those with CKD were more likely to be sent to rehabilitation facilities which could indirectly increase healthcare costs. Some limitations of our study may include a relatively smaller sample size and challenges with accurate identification of diagnosis and complications with ICD codes.

## Conclusions

Overall, CKD confers significant morbidity and mortality to patients undergoing TEER. There are higher rates of cardiovascular morbidities, including heart failure, cardiogenic shock, and cardiac tamponade. Additionally, respiratory complications, AKI, and the need for dialysis are rampant in this patient group. Financial implications are not excluded, with CKD patients having prolonged hospital stays and associated high healthcare costs. Providers should be aware of these discrepancies in outcomes for patients with CKD in need of TEER to help better optimize their care.
